# Compensation-free high-dimensional free-space optical communication using turbulence-resilient vector beams

**DOI:** 10.1038/s41467-021-21793-1

**Published:** 2021-03-12

**Authors:** Ziyi Zhu, Molly Janasik, Alexander Fyffe, Darrick Hay, Yiyu Zhou, Brian Kantor, Taylor Winder, Robert W. Boyd, Gerd Leuchs, Zhimin Shi

**Affiliations:** 1 Department of Physics, University of South Florida, Tampa, FL USA; 2 College of Natural Science, Michigan State University, East Lansing, MI USA; 3 The Institute of Optics, University of Rochester, Rochester, NY USA; 4 Department of Physics, University of Ottawa, Ottawa, ON Canada; 5 Max Plank Institute for the Science of Light, Erlangen, Germany

**Keywords:** Atmospheric optics, Fibre optics and optical communications

## Abstract

Free-space optical communication is a promising means to establish versatile, secure and high-bandwidth communication between mobile nodes for many critical applications. While the spatial modes of light offer a degree of freedom to increase the information capacity of an optical link, atmospheric turbulence can introduce severe distortion to the spatial modes and lead to data degradation. Here, we demonstrate experimentally a vector-beam-based, turbulence-resilient communication protocol, namely spatial polarization differential phase shift keying (SPDPSK), that can reliably transmit high-dimensional information through a turbulent channel without the need of any adaptive optics for beam compensation. In a proof-of-principle experiment with a controllable turbulence cell, we measure a channel capacity of 4.84 bits per pulse using 34 vector modes through a turbulent channel with a scintillation index of 1.09, and 4.02 bits per pulse using 18 vector modes through even stronger turbulence corresponding to a scintillation index of 1.54.

## Abstract (web summary)

Resistance to turbulence is an ongoing challenge for point-to-point freespace communications. Here the authors present a protocol for encoding a large amount of information in vector beams that are transmittable through a moderately strong turbulent channel without adaptive beam compensation.

## Introduction

Free-space optical communication offers flexibility, security, and large-signal bandwidth as compared to other means of communication^1–3^. Recently, there has been a great amount of research interest in using spatially structured light for optical communication^4–6^ as the spatial modes provide a new degree of freedom to encode information, thereby greatly increasing the system capacity and spectral efficiency within a finite spatial bandwidth of an optical channel. Among various families of spatial modes that have been investigated, the orbital angular momentum (OAM) modes of light have been used most widely and successfully to increase the information capacity of a free-space optical link^7–11^. However, atmospheric turbulence can impose serious practical limitations on the utilization of OAM or other spatial modes, as the fluctuation in the refractive index of air can alter both the transverse amplitude and phase structures of OAM modes. This, in turn, leads to crosstalk between neighboring OAM modes and degradation of the information capacity of the free-space optical link^12–16^. Adaptive optics has been used as the standard approach to compensate for distortions caused by mild or thin turbulence^17–19^, yet it remains a challenge to compensate for beam distortions caused by strong or volumetric turbulence. Other approaches such as image recognition based on artificial intelligence and machine learning^20–23^ have also been demonstrated to resolve the information encoded in severely distorted structured beams through turbulence. However, these approaches require the slow acquisition of images and significant computing resources, making them unsuitable for high-speed operation in real time.

Meanwhile, vector beams^24^ are optical fields that possess non-uniform spatial profiles in both their complex amplitude and polarization. The diversity in degrees of freedom within the vectorial optical fields has brought new dimensions for fundamental studies^25–27^ and led to new optical applications^28–36^ with performances surpassing those of conventional approaches. In particular, it has been shown that atmospheric turbulence is polarization insensitive, and, therefore, the spatial polarization profiles of vector beams are more resilient to atmospheric turbulence when compared to the transverse phase profiles^37–39^. Interestingly, many previous studies^34,39–43^ have shown that vector beam-based protocols do not outperform their scalar beam-based counterparts, and that both are equally vulnerable to atmospheric turbulence. Thus, it remains a challenge to effectively utilize a large number of spatial modes to transmit information through a turbulent channel.

In this work, we propose a new high-dimensional communication protocol, namely, spatial polarization differential phase shift keying (SPDPSK), that encodes and decodes high-dimensional information based on orthogonal spatial polarization states of a family of vector vortex beams. We observe experimentally that the spatial polarization profile of vector vortex beams can be resilient against atmospheric turbulence by using a carefully designed detection scheme. By utilizing such advantages, our SPDPSK protocol can transmit high-dimensional information reliably through a moderately strong turbulence cell with a scintillation index of up to 1.54 in the absence of any beam compensation mechanism. We demonstrate a proof-of-principle, high-dimensional communication system by transmitting 34 information levels (5.09 bits of information) per pulse through a free-space channel in the moderately strong turbulence regime with small information loss. We emphasize that, to the best of our knowledge, no effective high-speed OAM communication protocol has been experimentally realized under such turbulence strength.

## Results

### Principle

**Fig. 1:**
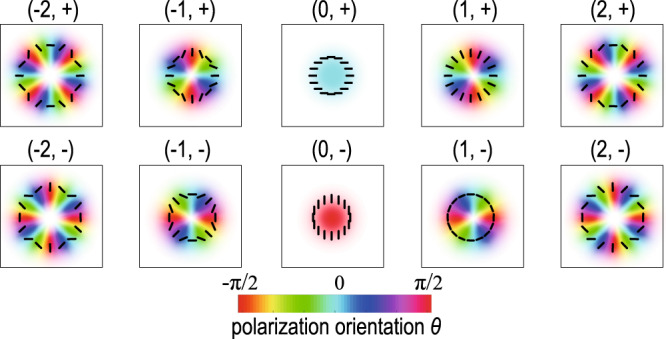
Spatial profiles of vector beams. The spatial polarization profiles of ten vector beams comprised of Laguerre–Gaussian (LG) beams in the circular polarization basis with opposite OAM charges along with 0 or *π* relative phase difference. The color indicates the polarization orientation while the color saturation indicates the beam intensity. The black lines indicate the local polarization state across the beams.

We here propose to use a family of vector vortex beams with orthogonal spatial polarization profiles to represent a large number of information levels. As an example, we consider a family of vector vortex beams, each formed by superposing two Laguerre–Gaussian (LG) beams that possess OAM charges of opposite signs in the two circular polarization bases. The *m*th order LG vector vortex beam can be expressed as follows:

1 $${\mathbf{E}}_{m,\pm }(r,\theta ,z) = {\hat{ e}}_{\ell }{{\rm{LG}}}_{0,m}(r,\theta ,z)\pm {\hat{ e}}_{r}{{\rm{LG}}}_{0,-m}(r,\theta ,z),$$

where LG_0,*m*_ denotes the spatial field profile of a Laguerre–Gaussian beam with radial index *p* = 0, azimuthal index *m*, and the subscript + or − indicates the relative phase difference of 0 or *π* between the two polarization components. In this configuration, allowing the mode order index *m* to possess *N* different values, we can have a total number of 2*N* orthogonal vector vortex beams to represent 2*N* information levels. Figure 1 shows the spatial polarization profile of ten such vector vortex beams with mode order index, *m* = −2, −1, 0, 1, 2, and with a relative phase difference of 0 and *π* between the two circular polarization components. These ten vector vortex beams represent ten different information levels in our protocol.

The vector vortex beams then propagate through a free-space optical link. At the receiver end, the beam is first split into *N* copies. Each copy then passes through a decoding channel designed for the identification of the *n*th order vector modes. In each decoding channel, the beam first passes through an anisotropic decoding phase plate with a differential phase response of Δ*ϕ*_*n*_(*r*, *θ*) = 2*nθ* between the right- and left-handed circular polarization components. The decoded beam then passes through a polarizing beam splitter (PBS), and subsequently, a balanced detector is used to measure the power difference between the separated H and V polarization components. When an *m*th order vector vortex beam passes through an *n*th order decoding channel, the normalized detected signal is given by

2 $${P}_{m,\pm }^{n}=\left\{\begin{array}{ll}\pm\! 1&n=m\\ 0&n\,\ne\, m\end{array}\right..$$

**Fig. 2:**
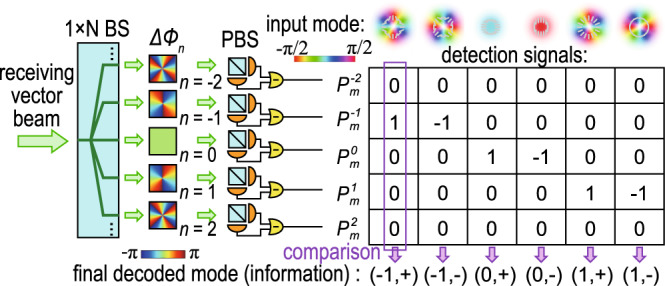
The principle of signal detection. The incoming optical signal is split into *N* copies, each passing through an *n*th order polarization-dependent, decoding phase mask before the differential power between H and V polarization is measured. Here, the color represents the differential phase profile of each decoding mask. All *N* decoded signals are then compared to determine the final detected information level (vector beam modes). The table on the right shows the detected signals of five decoding channels for six input modes (shown on the top with the color representing the polarization orientation), and the final decoded information level at the bottom.

As can be seen, for the *n*th order decoding channel, an incoming *n*th order vector beam would result in a detection signal of 1 or –1 for the (*n*, +) and (*n*, −) input modes, respectively. On the other hand, an incoming *m*th order vector vortex beam would result in a detection signal of 0 if *m* ≠ *n*. All *N* differently decoded detection signals for the same received beam are then compared to determine its information level. An illustration of the detection process for the six lowest-order vector beams with five decoding channels is shown in Fig. 2. Since the information is encoded in the spatial polarization profile of the beam, which is determined by the spatially-varying phase difference between the two polarization components of the vector beam, we name our protocol spatial polarization differential phase shift keying (SPDPSK). Note that several previous works have also used orthogonal vector beams to represent high-dimensional information^34,39–43^. Yet, our decoding scheme is vastly different from all previous approaches including image recognition and vector-beams to OAM-beam conversion followed by OAM detection. Our decoding scheme, which detects the relative phase difference between the two polarization components, plays a key role in achieving significantly improved performance in transmitting information through turbulence.

When the vector beam propagates through a turbulent channel, both circular polarization components of the beam can experience severe phase and amplitude distortions. However, because the atmospheric turbulence is insensitive to the polarization, the relative difference in the distortion between the two polarization components, which determines the distortion of the spatial polarization profile, tends to be much smaller than the distortion of the complex-field profile of each polarization component. Since the information is encoded in the spatial polarization profile rather than the complex-field profile of the beam, it is better conserved under turbulent conditions. The turbulence resilience of our SPDPSK protocol can also be understood analogously to the well-known differential phase shift keying (DPSK) protocol being resilient to phase fluctuation in optical fibers. More detailed descriptions of the theoretical framework of our protocol are given in Supplementary Note 1.

### Experimental results

**Fig. 3:**
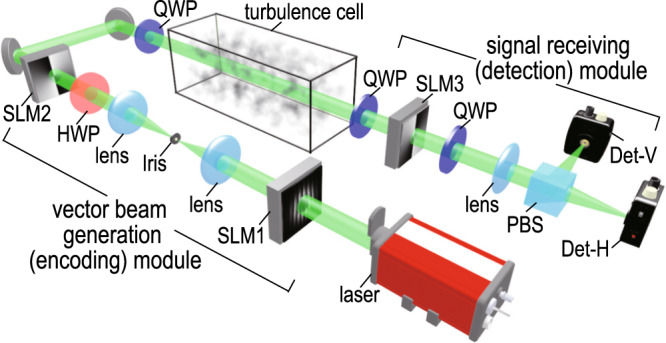
Schematics of the experiment. The schematic diagram of the proof-of-principle experiment including a vector beam generation module, a controllable turbulence cell, and a signal detection module. SLM spatial light modulator, HWP half-wave plate, QWP quarter-wave plate, PBS polarizing beam splitter, Det detector.

To examine the performance of our SPDPSK protocol under various atmospheric turbulence conditions, we construct a proof-of-principle experiment with a controllable turbulence cell. As illustrated in Fig. 3, we use two cascaded, phase-only spatial light modulators (SLMs) with polarization optics to generate the desired vector vortex beams. The generated beam is expanded using a 3.3X telescope and sent through a hotplate-based turbulence cell, wherein the strength of turbulence can be controlled by adjusting the hotplate temperature. At the receiving end, the beam is demagnified by another 3.3X telescope before propagating through one decoding channel. The beam is reflected off from a third SLM (SLM3 in Fig. 3), which is used as the anisotropic decoding phase plate. The decoded beam then passes through a polarizing beam splitter (PBS), and the powers of the separated horizontal and vertical components of the decoded beam are measured by two photodetectors. The two power readings are then subtracted to give the final detection signal of the current decoding channel.

**Fig. 4:**
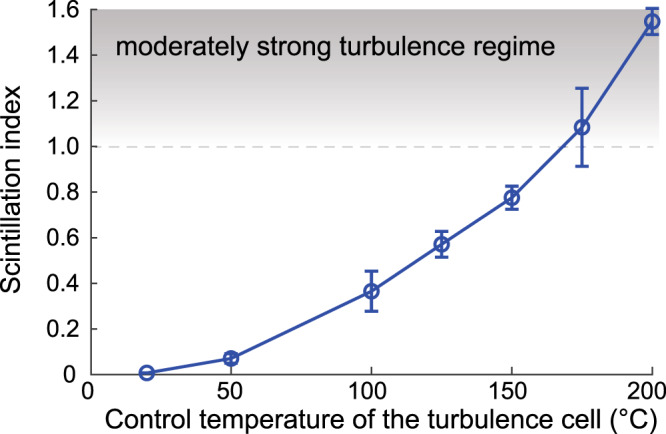
Measured scintillation index. Measured scintillation index of Gaussian beams propagating through the turbulence cell as a function of the control temperature. Error bars represent standard deviation over three independent measurements.

We first characterize the turbulence strength of our free-space channel by propagating a large Gaussian beam through the channel. Moderate or strong turbulence is known to cause random beam distortion and beam wandering, both of which can cause large fluctuations of the beam intensity at a given point on the receiver plane. Such fluctuations can be quantitatively evaluated using the scintillation index defined by the following^44,45^:

3 $${\sigma }_{I}^{2}=\frac{\langle {I}^{2}\rangle }{{\langle I\rangle }^{2}}-1,$$

where 〈⋅〉 denotes ensemble average, and *I* is the measured beam intensity at the statistical center of the beam. Here, we measure the scintillation index of a large Gaussian beam at the decoding SLM3 plane after it propagates through our turbulent channel. The measured scintillation index as a function of control temperature of the hotplate is shown in Fig. 4. It can be seen that the measured scintillation index value ranges from ∼0 to 1.54, which indicates that our setup can provide up to moderately strong turbulence^44^ that is controllable and adjustable. For realistic urban environments where the refractive index structure parameter, ${C}_{n}^{2}$, typically is of the order of 10^−13^ to 10^−15^, the maximum scintillation index value of 1.54 obtained in our experiment corresponds to a maximum propagation length in the range of 1 to 20 km according to our numerical simulation using the modified von Karman turbulence model. Note that both beam distortion and beam wandering contribute to an increase of scintillation index, and, therefore, the scintillation index has been used as a standard figure of merit to quantify the strength of the turbulence. Note that in a real free-space optical channel with an optical path length on the order of kilometers, the divergence of Laguerre–Gaussian beams due to diffraction and turbulence-induced beam wandering are also common challenges that can contribute to the degradation in the detected signals. In our experiment, the aperture of our optics is approximately twice the size of the largest beams (± eighth-order vector beams) at the receiving end in a turbulence-free case, and, therefore, the effect of beam clipping due to the limited aperture size is relatively weak in our experiment. In realistic kilometer-long experiments, the beam divergence can be minimized by optimizing the radial profile of the beams^46^, and the beam wandering can be significantly reduced by using tilt-and-pitch correction, which is much faster and significantly less expensive when compared to full wavefront compensation.

**Fig. 5:**
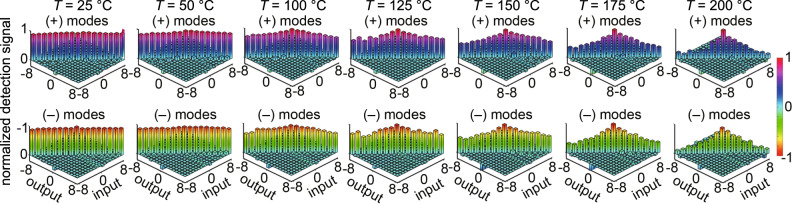
Measured signal matrices. The average values of the directly measured signal of each input mode through each decoding (output) channel at various turbulence strengths. The color indicates the normalized signal value between −1 and 1. The top and bottom rows are for (+) and (−) input modes as described in Eq. (1), respectively.

In our communication experiment, we use 17 mode orders (*m* = −8 to 8), which enables a total of 34 information levels, i.e., 5.09 bits of information per pulse. For each transmitted mode at a given turbulence strength, we measure the detection signals through each decoding channel in sequence. A total of 2500 measurements are taken for each encoding–decoding configuration, and the averaged detection signal matrices for all the (+) and (−) input modes at various turbulence levels are shown in Fig. 5. As can be seen from the figure, when the channel is turbulence-free, the correctly decoded signals of all 17 mode orders (the diagonal elements) are ∼1 (or –1) for the (+) and (−) modes, respectively. Meanwhile, the incorrectly decoded detection signals (the off-diagonal elements) remain very small and close to zero. As the turbulence strengthens, the average values of the correctly decoded signals gradually drop, more rapidly in the case of higher mode orders. This is due to higher-order modes exhibiting a larger difference in the wavefronts of the two polarization components, which leads to a larger difference in the distorted transverse profile as they co-propagate through the turbulence. The incorrectly decoded signals can fluctuate with either positive or negative values, and, therefore, their average values remain near zero even at the strongest turbulence level in our experiment as shown in the off-axis elements in Fig. 5. In general, the fluctuations of all the detected signals increase at higher turbulence strengths and for larger mode orders.

**Fig. 6:**
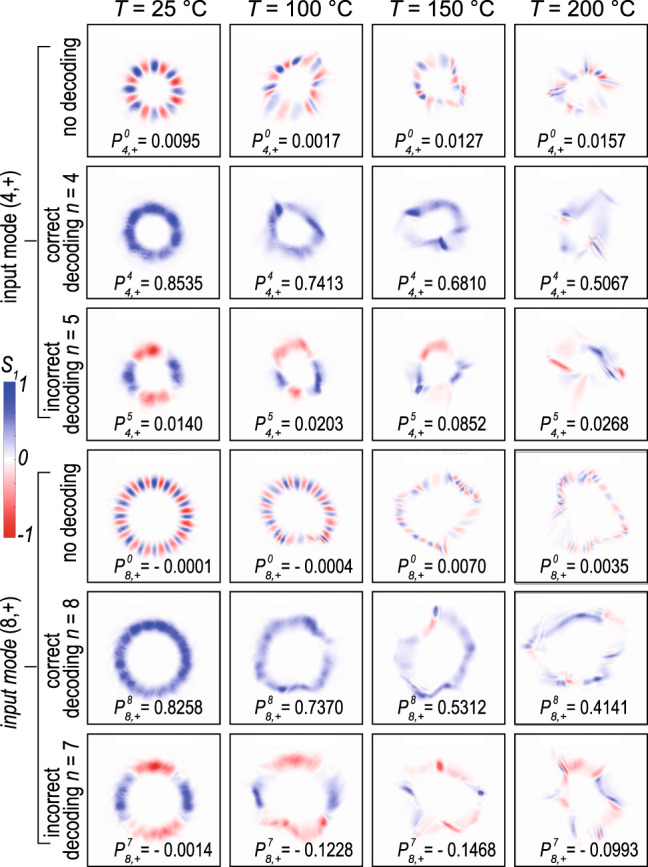
Recorded polarization profiles through different decoding modules. The measured *S*_1_ Stokes parameter profile of input (*m* = 4, +) (upper) and (*m* = 8, +) (lower) vector vortex modes propagating through the free-space channel at various turbulence settings with no decoding, correct decoding, and incorrect decoding, respectively. The color indicates the value of the normalized *S*_1_ parameter between −1 and 1. More snapshots of the profiles can be found in Supplementary Movie 1 for input mode (*m* = 4, +) and Supplementary Movie 2 for input mode (*m* = 8, +).

To gain a more direct understanding of the preservation and eventual degradation of the spatial polarization profiles through turbulence, we use a polarization-resolving camera to capture the spatial polarization profiles of two representative vector vortex beams, (*m* = 4, +) and (*m* = 8, +), after propagating through the free-space channel with various turbulence strengths and with no decoding, correct decoding, and incorrect decoding, respectively. From the no-decoding results, it can be seen that while the intensity profile of the vector beams experiences greater distortion at stronger turbulence levels, the polarization profile is, in fact, much better preserved. As a result, when the beam is decoded correctly, the vector beam is transformed into a scalar one that can lead to a large detection signal. Note that the detection signal listed below each Stokes parameter, *S*_1_, profile in Fig. 6 is calculated by summing over the *S*_1_ profile directly captured by the polarization-resolving camera with a relatively low polarization extinction ratio, leading to lower detection signal values as compared to the results shown in Fig. 5 that are obtained by a PBS and two photodetectors. As the channel enters into the moderately strong turbulence regime, i.e., a scintillation index value larger than 1 that corresponds to the hotplate control temperature, *T* > 150 ^∘^C, the polarization profiles of the transmitted vector beams experience more significant distortions resulting in the decoding becoming less effective. Furthermore, the polarization profiles of higher-order vector modes become distorted more strongly, which explains the more rapid drop in their correctly-decoded detection signals as shown in Fig. 5. On the other hand, when the beam is decoded incorrectly with a mismatching polarization phase mask, the power of the beam is still well split between the two polarizations as indicated by equally distributed blue and red colors. This provides an explanation as to why the incorrectly decoded detection signals are all close to zero, with the largest non-zero average values occurring at *n* = *m* ± 1.

**Fig. 7:**
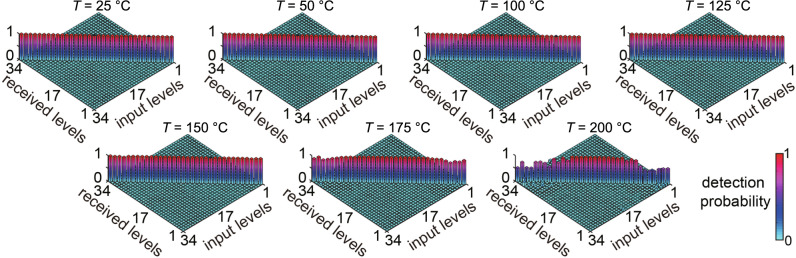
Experimentally obtained detection probability matrices. The experimentally measured detection probability matrix for the transmitted and received information levels at different turbulence strengths. The horizontal axes denote the encoded and received information levels, and the color indicates the value of the detection probability between 0 and 1.

The final information level of each received beam is determined by selecting the largest positive (or negative) value among the 17 detection signals, each through a different decoding channel. Note that in a full-scale system, all detection signals should be obtained simultaneously for each incoming beam. However, our numerical simulation indicates that the fluctuations in the detection signals of different decoding channels for each given order of incoming vector beam are quite uncorrelated. This can be understood as a consequence of the orthogonality of the spatial polarization modes or the spatial mode diversity of the vector modes^47,48^. Thus, as a proof-of-principle demonstration, we perform the signal detection in different decoding channels sequentially and then compare them to determine the received information level. The obtained information detection probability matrices for all of the 34 information levels are shown in Fig. 7. As can be seen, the probability of correctly detecting the information levels remains approximately unity even when the control temperature of the turbulence cell is 175 ^∘^C, corresponding to a scintillation index of 1.09. As the temperature increases, the probability of modes being incorrectly detected as the neighboring modes generally increases and is more severe for higher mode orders.

Based on the experimentally measured detection probability matrix, we further compute the average optical signal error rate (ER) and the channel mutual information (MI) according to the following formulas:

4 $${\rm{ER}}(2N)=\mathop{\sum }\limits_{\alpha =1}^{2N}{P}_{\alpha }\sum _{\beta \ne \alpha }{P}_{\beta | \alpha },$$

5 $${\rm{MI}}(2N)=	\, -\mathop{\sum }\limits_{\alpha =1}^{2N}{P}_{\alpha }{\mathrm{log}\,}_{2}({P}_{\alpha })\\ 	\, +\mathop{\sum }\limits_{\alpha =1}^{2N}{P}_{\alpha }\sum _{\beta \ne \alpha }{P}_{\beta | \alpha }{\mathrm{log}\,}_{2}({P}_{\beta | \alpha }),$$

where 2*N* is the total number of vector modes used, and *P*_*α*_ is the probability of information level *α* in the encoded data stream. Here, we assume all levels are equally probable in the encoded data stream such that *P*_*α*_ = 1/(2*N*), and *P*_*β*∣*α*_ is the conditional probability of detecting an incoming information level *α* as information level *β*.

**Fig. 8:**
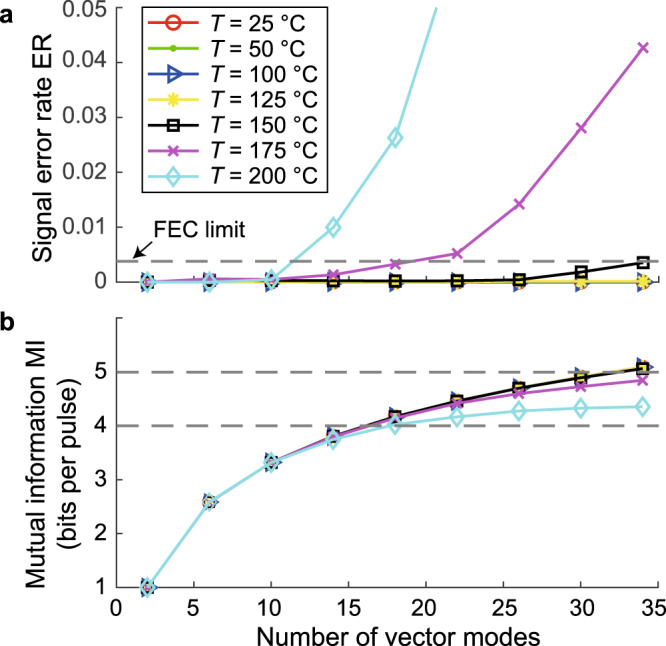
Experimentally obtained transmitted signal quality. The experimental (**a**) signal error rate and (**b**) mutual information as functions of the number of vector modes used in the system at various temperatures. The forward error correction (FEC) limit of 3.8 × 10^−3^ is plotted as a dashed line. Here, all levels are considered equally probable in the data stream.

Figure 8 shows the average optical signal error rate, ER, and the channel mutual information, MI, as functions of the number of vector modes used in the system at various temperatures (turbulence levels). As can be seen, the system is practically error-free in weak and moderate turbulence conditions (corresponding to scintillation index values up to 0.8 or temperatures up to 150 ^∘^C), with an average signal error rate less than 0.35%, which is lower than the forward error correction (FEC) limit^49^ of 0.38%. The mutual information between the sender and the receiver, which describes the channel capacity, follows the theoretical upper bound of ${\mathrm{log}\,}_{2}(N)$ bits per pulse, which corresponds to 5.09 bits per pulse for 34 vector modes. As the turbulence strengthens and the scintillation index becomes larger than one, the error rate starts to increase, owing largely to the degradation in the spatial polarization profile of higher-order modes. For a system using 34 vector modes, the average error rate increases to 4.3% and 19.7% at temperatures of 175 ^∘^C and 200 ^∘^C, respectively. This corresponds to a channel mutual information of 4.84 and 4.35 bits per pulse, respectively. However, if one reduces the total number of vector modes to 18, the average error rate is reduced significantly to only 2.6% at the highest temperature setting of 200 ^∘^C with a scintillation index of 1.54. This corresponds to a channel capacity of 4.02 bits of information per pulse, only slightly lower than the theoretical upper limit of 4.17 bits-per-pulse for an 18-level system.

**Fig. 9:**
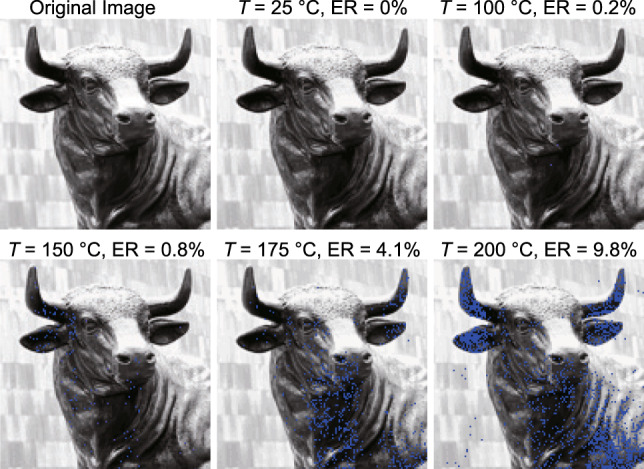
Transmitted image data. The retrieved 5-bit grayscale image encoded in a 32-level SPDPSK system sent through a free-space channel with various turbulence strengths. The blue pixels indicate the incorrect data received, and the error rate of each image is listed on the top.

Lastly, we demonstrate the transfer of a data packet through the turbulent channel using our SPDPSK communication protocol. The data packet used here is a 5-bit grayscale image composed of 128 × 128 pixels. We use 32 non-zeroth-order vector modes (see Supplementary Note 2 for details of the encoding table) such that the 5-bit grayscale information of each pixel is fully encoded into one pulse. The received images at various turbulence strengths are shown in Fig. 9, in which the pixels whose gray level information is received incorrectly are marked in blue. It can be seen that our high-dimensional communication system can reliably transmit the image through a free-space channel with up to moderately strong turbulence. Additionally, the error rate measured in the image data matches well with our previous results of the detection probability.

In summary, we have introduced a spatial polarization differential phase shift keying (SPDPSK) communication protocol that encodes high-dimensional information data onto the spatial polarization profile of an optical beam. We have shown experimentally that the spatial polarization profile of vector vortex beams can be resilient to moderately strong atmospheric turbulence, and, therefore, the SPDPSK protocol can transmit high-dimensional data without the need of any beam compensation mechanism. Using 34 orthogonal vector vortex beams, we have measured a channel capacity of 4.84 bits of information per pulse through a turbulent channel with a scintillation index of 1.09. When the scintillation index was increased to 1.54, we successfully used 18 vector modes to effectively transmit 4.02 bits of information per pulse. To further improve the system performance, one can consider using minimum energy loss modes^46^ to form the vector modes, incorporating a pitch-and-tilt correction mechanism in the detection module to reduce the effect of beam wandering, or using multiple-input and multiple-output (MIMO) equalization algorithms to further mitigate crosstalk^50–53^. Our SPDPSK protocol paves the way towards a practical and robust solution for high-capacity, free-space communication under natural, harsh environments.

## Methods

To generate the desired *m*th order vector vortex beam, a laser beam from a 532-nm laser (Coherent Compass M315) with horizontal polarization is first expanded, collimated, and launched onto a phase-only spatial light modulator (SLM1; Cambridge Correlators SDE1024). A computer-generated hologram (CGH) for LG_0,*m*_ is imprinted onto SLM1^54^, and the diffracted light passes through a 4-*f* imaging system with spatial filtering at the focal plane. The transmitted light in the first diffraction order is adjusted to be 45 degrees polarized before it reaches a second phase-only SLM (SLM2; Hamamatsu) placed at the image plane, which is also responsive only to horizontal polarization. SLM2 is imposed with a phase profile of −2*mθ* or −2*mθ* + *π* for the (+) and (−) modes, respectively. As a result, the horizontal and vertical components of the resultant beam share identical amplitude profiles of a Laguerre–Gaussian mode but with opposite OAM charges and an overall relative phase shift of 0 or *π*. The beam further passes through a quarter-wave plate to convert the H and V polarizations into left- and right-handed circular polarizations, respectively, resulting in the desired *m*th order vector vortex beams, ***E***_*m*,±_.

The generated vector vortex beam is then expanded by a 3.3X telescope (Keplerian type with two lenses of focal lengths 15 and 50 cm, respectively) before it propagates through a hotplate-based turbulence cell ∼60 cm in length. The strength of the turbulence is controlled by adjusting the temperature of the hotplate. The transmitted beam then passes through a receiving telescope with 3.3X demagnification and a quarter-waveplate before being launched onto a decoding spatial light modulator (SLM3; Hamamatsu). The decoding SLM3 responds only to horizontal polarization, and is imposed with a spiral phase profile of Δ*ϕ*_*n*_ = 2*nθ* for the *n*th order decoding channel. The decoded beam then passes through a quarter-waveplate and a polarizing beam splitter, and the two outputs are focused onto two photodetectors.

Note that in a full-scale system, the vector beam that reaches the receiving end should first pass through a 1-to-*N* beam splitter, resulting in *N* identical copies, where *N* is the total order number of vector modes used. Each copy would then pass through a decoding module designed for the identification of the *n*th order vector mode, and a total of *N* final detection signals would be obtained simultaneously for each incoming pulse. The information (spatial polarization mode) of the received signal is then determined by comparing these *N* signals. However, our numerical simulation indicates that the fluctuations in these *N* signals for any input mode due to turbulence can be considered uncorrelated. Such lack of correlation in the fluctuations may also be interpreted as a consequence of the spatial mode diversity for the modes that we use in our protocol^47,48^. Therefore, our experimental demonstration of collecting the detection signal of one decoding channel at a time is still valid in assessing the data fidelity and error rate with a similar performance to a full-scale system.

## Supplementary information

Supplementary Information

Description of Additional Supplementary Files

Supplementary Movie 1

Supplementary Movie 2

## Data Availability

The data that support the findings of this study are available from the authors on reasonable request.
