# Supplementary material for: Compensation-free high-dimensional free-space optical communication using turbulence-resilient vector beams
Source: Nat Commun. 2021 Mar 12;12:1666. doi: 10.1038/s41467-021-21793-1 (PMC7955115; doi:10.1038/s41467-021-21793-1)
Supplement: Supplementary file 1 — Supplementary Information [file 41467_2021_21793_MOESM1_ESM.pdf]

Compensation-free high-capacity free-space optical communication using turbulence-resilient vector beams: supplementary material

Ziyi Zhu[1], Molly Janasik[1,2], Alexander Fyffe[1], Darrick Hay[1], Yiyu Zhou[3], Brian Kantor[1], Taylor Winder[1], Robert W. Boyd[3,4], Gerd Leuchs[5], and Zhimin Shi[1,*]

[1] *Department of Physics, University of South Florida, Tampa, FL 33620, USA*

[1] *College of Natural Science, Michigan State University, East Lansing, MI 48824, USA*

[3] *The Institute of Optics, University of Rochester, Rochester, New York 14627 USA*

[4] *Department of Physics, University of Ottawa, Ottawa, ON K1N 6N5 Canada*

[5] *Max Plank Institute for the Science of Light, Erlangen, Germany*

[*] *zhiminshi@usf.edu*

1 Supplementary Note 1

We here describe in detail the theoretical framework of our SPDPSK protocol. As an example, each vector vortex beam can be formed by a superposition of two Laguerre-Gaussian (LG) beams that possess OAM charges of opposite signs in the two circular polarization bases along with a relative phase difference of 0 or π. Such a LG vector vortex beam can be expressed as follows:

$$\begin{aligned}\mathbf{E}_{m,\pm}(r,\theta,z) &= \hat{e}_\ell E_{\ell,m,\pm}(r,\theta,z) + \hat{e}_r E_{r,m,\pm}(r,\theta,z) \\ &= \hat{e}_\ell \mathrm{LG}_{0,m}(r,\theta,z) \pm \hat{e}_r \mathrm{LG}_{0,-m}(r,\theta,z), \end{aligned} \tag{S1}$$

where $\mathrm{LG}_{p,l}$ denotes the spatial field profile of a Laguerre-Gaussian beam

$$\begin{aligned}\mathrm{LG}_{p,l}(r,\theta,z) &= \sqrt{\frac{2p!}{\pi(p+|l|)!}}\frac{1}{w(z)}\left(\frac{\sqrt{2}r}{w(z)}\right)^{|l|} e^{-\frac{r^2}{w(z)^2}} \\ & L_p^{|l|}\left(\frac{2r^2}{w(z)^2}\right) e^{-ik\frac{r^2}{2R(z)}} e^{il\theta} e^{-ikz} e^{i\phi(z)}, \end{aligned} \tag{S2}$$

and where p and l denote the radial and azimuthal index, respectively, $L_p^{|l|}(\cdot)$ is the generalized Laguerre polynomial, $w(z) = w_0\sqrt{1+(z/z_R)^2}$ is the beam waist, $z_R = \pi w_0^2/\lambda$ is the Rayleigh range and $R(z) =$

$z[1+(z_R/z)^2]$ is the radius of curvature of the beam. If m takes N different values, we have a total number of $2N$ orthogonal vector vortex beams to represent $2N$ information levels.

Since the spatial polarization profile is determined by the relative phase (and amplitude) of the two orthogonal polarization components, the information encoded in the spatial polarization profiles is essentially encoded as the difference of the spatially-varying phase profiles between the two polarization components. Thus, analogously to the well-known differential phase shift keying (DPSK) protocol in which the information is encoded in the relative phase between neighboring pulses in the time domain, we name our protocol spatial polarization differential phase shift keying (SPDPSK), indicating that the information is encoded in the relative phase between the two polarization components of a vector beam. In theory, the spatial polarization profiles can span a Hilbert space of infinitely large dimensions, indicating the number of information levels that can be encoded is infinitely large. In practice, the number of usable information levels is determined by the spatial-bandwidth product (Fresnel number) of the free-space optical link as well as the turbulence strength.

When the free-space optical channel is turbulence-free, the two polarization components of each vector vortex beam would pick up the same phase as the beam propagates through the channel; therefore the spatial polarization profile of the beam remains invariant upon propagation. Consequently, the spatially dependent phase difference between the two polarization components is independent of the propagation distance, which can be shown as follows:

$$\begin{aligned}\Delta\phi_{m,\pm}(r,\theta,z) &= \arg\left[E_\ell(r,\theta,z)\right] - \arg\left[E_r(r,\theta,z)\right] \\ &= 2m\theta + (0.5 \mp 0.5)\pi. \end{aligned} \tag{S3}$$

In order to decode the information at the receiver end, we first split the received vector vortex beam into N equal copies (see Fig. 1 in the manuscript). Each copy then passes through a decoding channel designed for the identification of the nth order vector modes. In each decoding channel, the beam first passes through an anisotropic decoding phase plate that has a polarization-dependent transmission function. The transmission function of the nth-order decoding phase plate can be written in the left- and right-handed circular polarization (LCP and RCP, respectively) bases as follows:

$$\mathbf{T}_n(r,\theta) = e^{i\phi_\ell(r,\theta)} \begin{bmatrix} 1 & 0 \\ 0 & e^{i\Delta\phi_n(r,\theta)} \end{bmatrix}, \tag{S4}$$

where $e^{i\phi_\ell(r,\theta)}$ is some transmission function for the left-handed circularly polarized light, and $\Delta\phi_n(r,\theta) =$

$2n\theta$ is the difference between the phase responses for the right- and left-handed circular polarization components. When a mth order vector vortex beam passes through such a nth order decoding phase plate, the transmitted field becomes

$$\begin{aligned} \mathbf{E}_{m,\pm}^{n,\text{out}} &= \mathbf{T}_n \mathbf{E}_{m,\pm}^{\text{in}} \\ &= \hat{e}_\ell \text{LG}_{0,m} e^{i\phi_\ell} \pm \hat{e}_r \text{LG}_{0,-m} e^{i\phi_\ell} e^{i2n\theta}. \end{aligned} \tag{S5}$$

When the output beam passes through a polarizing beam splitter (PBS), the intensity of the field exiting the two output ports becomes

$$\begin{aligned} I_{m,\pm}^{n,\text{h}} &= |\text{LG}_{0,m}|^2 \cos^2[(m-n)\theta - (1\pm 1)\frac{\pi}{4}], \\ I_{m,\pm}^{n,\text{v}} &= |\text{LG}_{0,m}|^2 \sin^2[(m-n)\theta - (1\pm 1)\frac{\pi}{4}]. \end{aligned} \tag{S6}$$

The normalized power of the two separated H- and V- components are given as follows:

$$\begin{aligned} P_{m,\pm}^{n,\text{h}} &= \begin{cases} \frac{1}{2} \pm \frac{1}{2} & n = m \\ 0.5 & n \neq m \end{cases}, \\ P_{m,\pm}^{n,\text{v}} &= \begin{cases} \frac{1}{2} \mp \frac{1}{2} & n = m \\ 0.5 & n \neq m \end{cases}. \end{aligned} \tag{S7}$$

The final nth order detection signal is obtained by taking the difference between the two above outputs, which is given by

$$P_{m,\pm}^{n} \equiv P_{m,\pm}^{n,\text{h}} - P_{m,\pm}^{n,\text{v}} = \begin{cases} \pm 1 & n = m \\ 0 & n \neq m \end{cases}. \tag{S8}$$

As one sees, for the nth order decoding channel, an incoming nth order vector beam would result in a detection signal of 1 or -1 depending upon the relative phase between the LCP and RCP components. On the other hand, an incoming mth order vector vortex beam would result in a detection signal of 0 if $m \neq n$ in the absence of turbulence.

2 Supplementary Note 2

To represent the 5-bit gray-scale information of each pixel in the picture, we use 32 vector vortex beams with order index, $m = -8, -7, ... -1, 1, ...7, 8$. The relation between each gray scale and the corresponding

vector vortex mode index is listed in table S1.

Table S1: 5-bit data encoding look-up table

gray level	0	1	2	3	4	5
mode index	(8,+)	(-8,+)	(8,-)	(-8,-)	(7, +)	(-7, +)
gray level	6	7	8	9	10	11
mode index	(7, -)	(-7, -)	(6, +)	(-6, +)	(6, -)	(-6, -)
gray level	12	13	14	15	16	17
mode index	(5, +)	(-5, +)	(5, -)	(-5, -)	(4, +)	(-4, +)
gray level	18	19	20	21	22	23
mode index	(4, -)	(-4, -)	(3, +)	(-3, +)	(3, -)	(-3, -)
gray level	24	25	26	27	28	29
mode index	(2, +)	(-2, +)	(2, -)	(-2, -)	(1, +)	(-1, +)
gray level	30	31				
mode index	(1, -)	(-1, -)				
