# Supplementary material for: Compensation-free high-dimensional free-space optical communication using turbulence-resilient vector beams
Source: Nat Commun. 2021 Mar 12;12:1666. doi: 10.1038/s41467-021-21793-1 (PMC7955115; doi:10.1038/s41467-021-21793-1)
Supplement: Supplementary file 2 — Description of Additional Supplementary Files [file 41467_2021_21793_MOESM2_ESM.docx]

**Description of Additional Supplementary Files**

File name: Supplementary Movie 1

Description: The measured S1 Stokes parameter profile of an input (m = 4,+) vector vortex mode propagating through the free-space channel at various turbulence settings with no decoding, correct decoding and incorrect decoding, respectively.

File name: Supplementary Movie 2

Description: The measured S1 Stokes parameter profile of an input (m = 8,+) vector vortex mode propagating through the free-space channel at various turbulence settings with no decoding, correct decoding and incorrect decoding, respectively.
